# Bridge symptoms between parenting styles and proximal psychological risk factors associated with adolescent suicidal thoughts: a network analysis

**DOI:** 10.1186/s13034-023-00674-z

**Published:** 2023-11-15

**Authors:** Wenwen Ou, Yumeng Yang, Yafei Chen, Yunjing Li, Siqi Yang, Yimei Lu, Liang Li, Mei Huang, Mohan Ma, Guanyi Lv, Xiaotian Zhao, Yaqi Qing, Yumeng Ju, Yan Zhang

**Affiliations:** 1 Department of Psychiatry, and National Clinical Research Center for Mental Disorders, The Second Xiangya Hospital of Central South University, Changsha, Hunan 410011 China; 2 Xiangya Medical School, The Second Xiangya Hospital, Central South University, Changsha, Hunan China

**Keywords:** Parenting styles, Risk factors, Suicidal ideation, Adolescence, Network analysis

## Abstract

**Background:**

Parenting styles and the associated proximal psychological factors are suggested to increase suicidal risks in adolescents. However, how the two factors interact and confer risks on the emergence of adolescent suicidal thoughts remains unclear. Herein, we used a network approach to investigate their interrelationship and explore whether the network properties predict adolescent suicidal thoughts.

**Methods:**

Self-report questionnaires were completed by 1171 students aged 12–16. Network analyses were performed by Gaussian graphical models estimating the adolescent psychosocial network structure of parenting styles and psychological variables including depression, anxiety, affective lability, rumination, and resilience. Furthermore, we re-examined the network by adding a variable measuring active suicidal thoughts. Moreover, we conducted linear regressions to examine the predictive utility of bridge symptoms for adolescent suicidal thoughts.

**Results:**

Resilience, Afraid, Rumination, Concentration, and affective lability (Anger) had the highest bridge strengths in the adolescent psychosocial network. Among the identified bridge symptoms, Resilience was negatively correlated with active suicidal thoughts (regularized edge weights = -0.181, bootstrapped 95% CIs: [-0.043, -0.155]), whereas affective lability (from Anxiety to Depression, Anger), Rumination, and Afraid were positively correlated with active suicidal thoughts, with edge weights (bootstrapped 95% CIs) ranging from 0.057 (0.001, 0.112) to 0.081(0.026, 0.136). Regression analysis showed that bridge strength was significantly correlated with active suicidal thoughts (R^2^ = 0.432, *P* = 0.001).

**Conclusion:**

Negative parenting styles may drive and maintain suicidal thoughts by modifying the key proximal psychological variables. Our findings highlight the important role of bridge symptoms, which may serve as vital targets for triggering adolescent suicide.

**Supplementary Information:**

The online version contains supplementary material available at 10.1186/s13034-023-00674-z.

## Introduction

Suicide remains a huge social and public health problem, causing almost one million deaths annually [1]. Notably, suicidal rates continue to rise from childhood to adolescence and peak during the adolescent period [2]. Moreover, suicide has been the second leading cause of non-natural death among children and adolescents worldwide [3]. A recent meta-analysis showed that the lifetime prevalence and 12-month prevalence of suicidal ideation in adolescents were 18.0% and 14.2%, respectively, and for suicidal attempts were 6.0% and 4.5%, respectively [4]. However, despite the alarming rates and detrimental effects of suicide among adolescents, few targeted interventions have been specifically established, and the efficacy was limited [5, 6]. A better understanding of the psychopathology of adolescent suicide is essential for developing effective prevention and early intervention strategies.

In recent years, numerous studies have uncovered the complex pathology of suicide, with multiple biological, environmental, and psychological factors proven to increase individuals’ vulnerability [7]. It was suggested that the emergence of adolescent suicidal thoughts could be traced back to negative parenting styles observed in childhood [8, 9]. Meanwhile, mounting evidence has shown that proximal correlates of suicidal behaviors primarily include depression, anxiety, affective lability, maladaptive rumination, and poor resilience [10–12]. These distal and proximal factors may contribute to prospective suicidal thoughts [13]. However, it has been unclear whether these factors lead to adolescent suicidal thoughts alone or in concert with each other, given that previous studies predominately focused on single risk factors. Moreover, some studies proposed that early distal risk factors (e.g., negative parenting) have been shown to trigger and maintain proximal vulnerabilities (e.g., high aversive emotions, high aversive cognitions, poor distress tolerance) for developing suicidal thoughts [14]. Yet, limited research has focused on which specific psychological variables activated by parenting styles might lead to the emergence of adolescent suicidal thoughts. A better understanding of the key factors in the interconnected chains linking parenting styles and adolescent suicidal thoughts would help reveal potential intervention targets in reducing the occurrence of adolescent suicide, especially for adolescents who underwent negative parenting.

Network analysis is an analytic method that focused on depicting symptom-level interrelationships graphically [15]. According to network theory, a psychological network can be parsimoniously represented by nodes and edges, wherein each node represents a psychological variable or symptoms, and each edge represents the correlation between two nodes. A node that highly connects two different symptom clusters is likely to increase the spread of symptoms from one cluster to another through the network; such nodes were conceptualized as bridge symptoms [16]. The activating and deactivating role of bridge symptoms have received great attention in psychiatric research; for example, a longitudinal study using network analysis found that the bridge symptoms between disruptive symptoms and internalizing symptoms in elementary school girls could predict the occurrence of future anxiety disorders in adolescence and early adulthood [17, 18]. Hence, targeting bridge symptoms might provide clinically meaningful information for the prevention and intervention prioritization of maladaptive behavior (i.e., suicidal behaviors) in adolescents [19, 20]. So far, several network studies examined the contributions of various risk factors to suicidal behaviors in veterans and young adults and revealed that a range of psychosocial variables conferred either protective or risky effects on suicidal ideation [21, 22]. However, the interplay of parenting styles and proximal psychological factors and their association with suicidal thoughts in adolescents is still not clearly understood and remains to be further explored.

Thus, we used the network analysis in a large sample of school-aged adolescents to investigate the critical psychological variables linking parenting styles and adolescent suicidal thoughts. We hypothesized that the “activated” bridge symptoms linking parenting styles and proximal psychological vulnerabilities exerted a distinct role in the development of suicidal thoughts. To test our hypothesis, we first constructed the symptom network to identify bridge symptoms between parenting styles and proximal psychological risk factors. Then, we investigated whether the identified bridge symptoms were significantly correlated with adolescent suicidal thoughts. In addition, we conducted linear regressions to examine the power of the bridge symptoms in predicting suicidal thoughts in adolescents.

## Method

### Participants and procedures

Participants (aged 12–16 years) were recruited via convenient sampling from 14 middle schools in 6 districts in Changsha Hunan Province, China. Students in grades 7 through 9 with parental consents to participate in our study and able to complete all scales and assessments independently were included in the study. Data were collected through a self-administered paper-based questionnaire anonymously. Participants were informed of relevant research information before the survey was started by well-trained psychiatrists. Moreover, we also provided extra mental health support during the survey if they had psychological distress or reported higher suicidal risk. This study was approved by the ethics committee of the Second Xiangya Hospital of Central South University. Written informed consents were obtained from the parents of all the participants.

The survey was conducted from February 12, 2021, to May 12, 2021, and in total, 1,501 participants completed the questionnaire survey. All answers were imported manually into the Electronic Data Capture System (EDC) of the Department of Psychiatry at the Second Xiangya Hospital by researchers. Questionnaire responses that had obvious errors were excluded.

### Measures

#### Demographic characteristics

Basic demographic information, including gender (male/female), age (years), residence (urban/rural), the only child in the family (yes/no), parental education level (Primary school or below/Middle school/High school/Bachelor/Master or above), and monthly household income per capita (estimated by Chinese yuan), were collected.

#### Parenting styles

Parenting styles were assessed by the short form of Egna Minnen Betraffande Uppfostran (s-EMBU) [23]. Each item in the questionnaire evaluated the perceived parenting styles of the father and mother, respectively. Items were scored by the Likert scale of 1–4 points (1 = “never” to 4 = “always”). The scale consists of three factors, including rejection (item 1,4,7,13,16,21), emotional warmth (item 2,6,12,14,19,23), and overprotection (item 3,5,8,11,17,18, 22). Negative parenting styles include rejection and overprotection from parents, whereas positive parenting styles refer to emotional warmth from parents. The s-EMBU demonstrated satisfactory psychometric properties among Chinese high school teenagers [24]. The s-EMBU also showed good internal consistency in our study with a Cronbach’s Alpha of 0.730 and 0.767 for the father and mother’s parenting styles, respectively.

#### Proximal psychological factors

Depressive and anxiety symptoms during the past two weeks in adolescents were measured by questionnaires of Patients Health Questionnaire-8 (PHQ-8) [25] and Generalized Anxiety Disorder-7 (GAD-7) [26], respectively. We excluded the item “Thoughts that you would be better off dead, or thoughts of hurting yourself in some way”, given that the last item in PHQ-9 overlapped the suicide measurement adopted in our study. Both PHQ-8 and GAD-7 have shown good reliability and validity in adolescents and college students [27, 28], The Cronbach’s α was 0.855 and 0.916 for PHQ⁃8 and GAD-7 in our study.

Affective lability during the last month was assessed by 18 items of the Affective Lability Scale (ALS-18) [29], which were grouped into three subscales measuring affective transitions from anxiety to depression (item 1,3,5,6,7), depression to elation (item 2,10,12,13,15,16,17,18), and anger (item 4,8,9,11,14), respectively. Each item was rated on a 4-point Likert scale of 0–3 points (0 = “very uncharacteristic of me” to 3 = “very characteristic of me”). A higher score indicates a higher level of affective lability. The Chinese version of ALS-18 demonstrated good psychometric properties among the Chinese student sample [30], This scale was tested to have good reliability in our sample (Cronbach’s α = 0.972).

Rumination during the last month was evaluated by a 10-item Ruminative responses scale (RRS-10) [31]. The scale is scored by summing the responses for each of the ten items, with each item rating from 1 to 4 (1 = “never” to 4 = “always”). A higher score suggests higher levels of rumination. The scale has demonstrated satisfactory psychometric properties among Chinese students [32]. The Cronbach’s α was 0.908 in our study.

Resilience during the last month was assessed using the 10-item Connor-Davidson Resilience Scale (CD-RISC-10) [33]. Items are rated on a 5-point Likert scale (0 = “not true at all” to 4 = “true nearly all of the time”), yielding a total score of 0 to 40. A higher score indicates better resilience. The CD-RISC-10 demonstrated satisfactory psychometric properties among Chinese adolescents [34]. The scale displayed good internal reliability in our sample (Cronbach’s α = 0.929).

#### Suicidal thoughts

Adolescent suicidal thoughts were measured by the 12-item concise health risk tracking self-report (CHRT-SR) [35] that evaluated a person’s active suicidal thoughts during the past 24 h. The CHRT-SR is composed of three subscales, including hopelessness (item 1, 3, 4, 5, 6, 7, and 8), social support (item 2 and 9), and active suicidal thoughts (item 10, 11, and 12). Given the factor of hopelessness and social support exhibiting high interrelations (Rp > 0.4) that termed “redundant nodes” in network analyses [36], we only included the factor of suicidal thought that assessed the severity of suicidal thoughts in the preceding 24 h. Participants rated each item on a scale from 1 to 5 (1 = “strongly disagree” to 5 = “strongly agree”), with higher values indicating more imminent suicidal risk. The CHRT-SR has demonstrated good internal reliability in adolescents with suicidality [37] and good internal reliability in our sample (Cronbach’s α = 0.923). Details of each questionnaire were provided (see Table S2).

### Statistical analysis

#### Data description and general differences

Data distribution was examined by probability plot and Kolmogorov-Smirnov test. Descriptive features of continuous variables with normal distribution were presented by mean and standard deviation (SD), non-normal variables by median and interquartile range (IQR), and categorical variables were presented by frequency and percentages. The differences in continuous variables with non-normal distribution between groups were examined by the Mann-Whitney U test on SPSS Version.25.0 software, with a significance threshold set at 0.05.

#### Adolescent psychosocial network

To show the interrelationships at symptoms levels between parenting styles and proximal psychological risk factors, firstly, we constructed the *adolescent psychosocial network* consisting of 26 nodes representing the parenting styles for father and mother (i.e., six items for rejection, emotional warmth, and overprotection from father and mother, respectively), emotional symptoms (i.e., eight items of PHQ-8, seven items of GAD-7, and three factors of ALS-18), and two items for rumination and resilience. The network structure was estimated by Gaussian graphical model (GGM) using Extended Bayesian Information Criterion (EBIC) selection via the R package “bootnet’’ and “qgraph” [38]. Specifically, we applied rank transformations (Spearman correlations as input) during the network construction considering the data were skewed [39]. Moreover, we created the layouts for each graph via the Fruchterman-Reinold algorithm [40], which prioritizes the more connected nodes in the center of the network and the less connected ones in the periphery. Before demonstrating network metrics, network accuracy and stability were assessed by bootstrapping procedure via the R package “bootnet’’. In addition, we tested the significance of the edge weights and centrality indices of each node.

Besides, we computed bridge strength in the established network. Bridge strength was calculated to denote the importance of a node to the network, as a recent study indicated that bridge closeness or betweenness may not be directly interpreted in the psychological network [41]. Bridge strength is calculated by the sum of absolute values of the edges connected to a given node to nodes in other symptom clusters, which denotes the importance of the symptom in connection with other symptom clusters [16].

#### Network associated with suicidal thoughts

To investigate whether the bridge symptoms were correlated with active suicidal thoughts in adolescents, we re-estimated the psychosocial network structure by including a continuous variable that measures active suicidal thoughts in adolescents over the past 24 h.

In light of a previous study reporting that girls who perceived more parent rejection and neglect in childhood tend to exhibit more suicidal attempts than boys [42], we also examined whether the network properties differ by gender. The difference in network properties including global strength, network structure, and edge weights via the R package “Network Comparison Test (NCT) [43]. The p-value was estimated by permutation test in NCT (5000 iteration), and the significance level was set at 0.05, 2-tailed.

Besides, to investigate the influence of demographic covariates on our original symptoms network, we reconstructed the network by including additional demographic factors (e.g., age, sex, residence, educational level, income). Then, the absolute differences in edge weights between the original symptom networks and the re-estimated network termed the “Delta network”, were calculated. The weaker difference in edge weights indicated that the covariates did not significantly affect the network structure.

#### Linear regression analysis

To further assess the predictive value of the identified bridge symptoms in the psychosocial network for active suicidal thoughts in adolescents, we conducted a linear regression analysis to explore the relationship between the bridge strengths of the psychosocial symptoms and their edge weights connected to active suicidal thoughts. The bridge strengths of each psychosocial symptoms were served as the independent variables and the edge weights connected the psychosocial symptoms to active suicidal thoughts were served as dependent variables. A significant correlation indicates that a node with higher bridge strength may predict higher suicidal thoughts.

## Results

### Demographic and psychological characteristics of the adolescents

A total of 1171 middle students were included in our sample. The sociodemographic characteristics of the current sample are summarized in Table 1. The mean age of our sample was 14.09 years old, and 660 (56.40%) were female. There were 795 participants (67.90%) living in urban areas, and 376 (32.10%) were the only child in the family. Descriptive data on all the proximal psychological factors and gender differences among participants’ scores were summarized (see Table S1). The median score of active suicidal thoughts was 6.00, with a significantly higher score in female adolescents than in male adolescents. Meanwhile, the median scores of PHQ-8 and GAD-7 were 7 and 5, respectively.

**Table 1:** Demographic characteristics

	Total sample (N = 1171)
**Age (Mean, SD)**	14.09(0.998)
**Gender (n, %)**	
Female	660 (56.4%)
Male	511 (43.6%)
**Residence (n, %)**	
Urban	795(67.9%)
Rural	376(32.1%)
**Only child (n, %)**	
Yes	376(32.1%)
No	795(67.9)
**Monthly household income per capita (n, %)**	
< 1000	40 (3.4%)
1000–5000	416 (35.5%)
5000–10,000	454 (38.8%)
10,000–50,000	233(19.9%)
> 50,000	28 (2.4%)
**Educational level of father (n, %)**	
Primary school or below	70 (6.7%)
Middle school	497 (42.4%)
High school	344(29.4%)
Bachelor	221(18.9%)
Master or above	39 (3.3%)
**Educational level of mother (n, %)**	
Primary school or below	89 (7.6%)
Middle school	510 (43.6%)
High school	347 (29.6%)
Bachelor	196(16.7%)
Master or above	29(2.5%)

* N: number of participants

### Adolescent psychosocial network

**Fig. 1:**
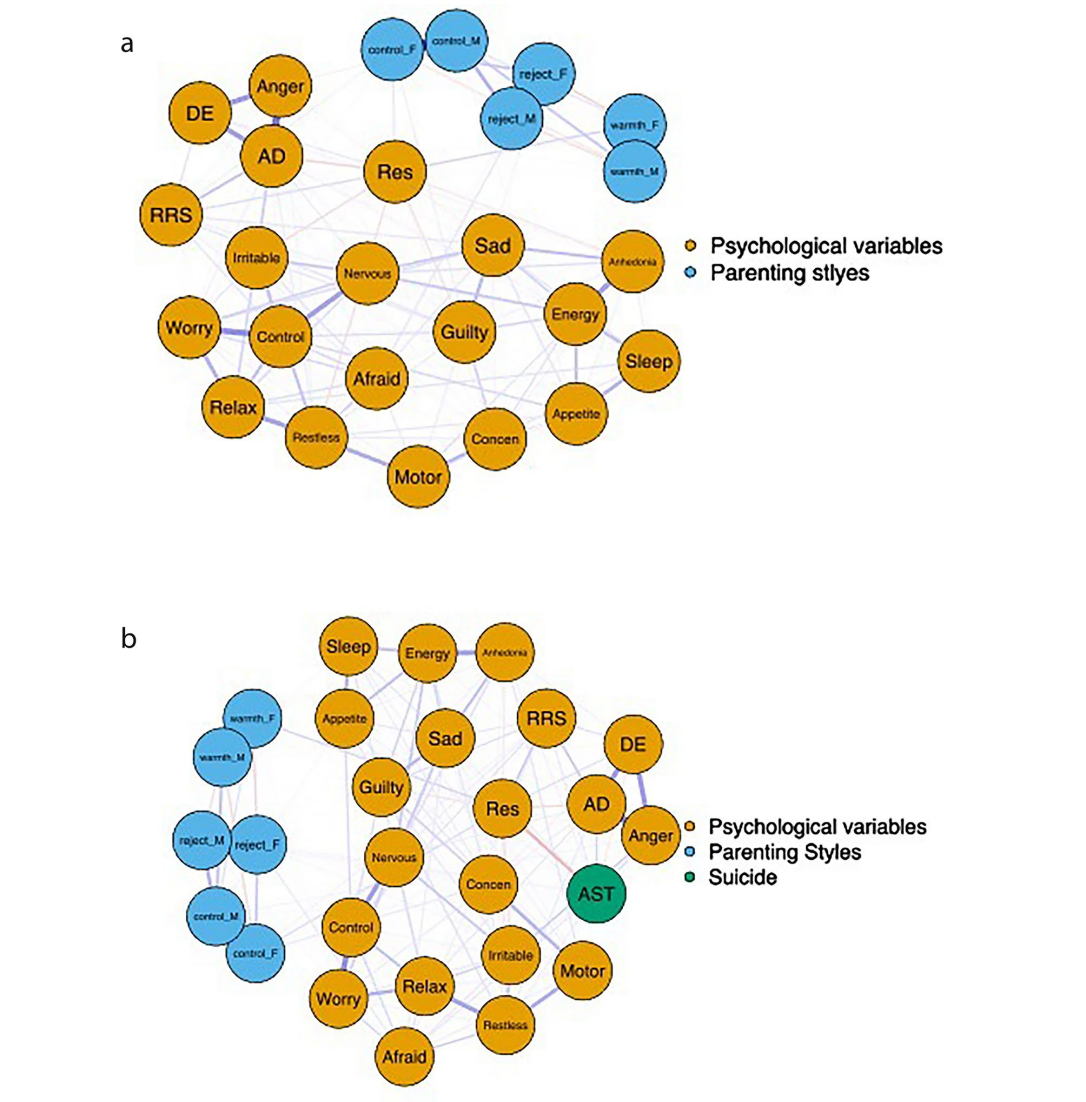
The estimated network structure of the adolescent psychosocial network (1a, left) and the network associated with suicidal thoughts (1b, right). The color of nodes represents different symptom clusters, orange = Psychological variables, blue = Parenting styles, green = Active suicidal thoughts. The thickness and saturation of edges indicate the absolute magnitude of correlation, and the color of the edges indicates the direction of the correlation (green represents a positive correlation, red represents a negative correlation). Node name and description see Table 2. For more detailed information of nodal representation please see Table S2

The symptom network of parenting styles and proximal psychological factors showed good network structure stability (see Figure S1). The adolescent psychosocial network structure is displayed in Fig. 1a. Estimated edge weights revealed correlations within all the proximal psychological factors, depression, anxiety symptoms, affective lability, and rumination being positively correlated with each other and negatively associated with resilience (see Table S3). Moreover, some proximal psychological factors were correlated with parenting styles. In terms of father’s parenting styles, “Afraid (afraid as if something would happen)” demonstrated a positive correlation with the father’s rejection; “Concentration (trouble concentrating on things)”, “Rumination”, and “Resilience” were positively correlated with the father’s overprotection; “Resilience” showed a positive correlation with the father’s emotional warmth. As for the mother’s parenting, “Anger (shift from normal mood to anger)” was positively correlated with the mother’s overprotection.

**Table 2:** Node name and symptom description

Symptom Cluster	Node Name (Abbreviation)	Item Description
Parenting styles	Reject_F Reject_M	Rejection From Father Rejection From Mother
	Warmth_F Warmth_M	Emotional Warmth From Father Emotional Warmth From Mother
	Control_F Control_M	Overprotection From Father Overprotection From Mother
Proximal psychological factors	Res	Resilience
	RRS	Rumination
	AD	Shift from anxiety to depression
	DE	Shift from depression to Elation
	Anger	Shift from normal mood to Anger
	Anhedonia	Anhedonia
	Sad	Sad Mood
	Sleep	Sleep
	Energy	Energy
	Appetite	Appetite
	Guilty	Guilty
	Concern	Concentration
	Motor	Motor
	Nervous	Nervous
	Control	Control Worry
	Worry	Worry A Lot
	Relax	Relax
	Restless	Restless
	Irritable	Irritable
	Afraid	Afraid
Suicidal thoughts	AST	Active suicidal thoughts

**Fig. 2:**
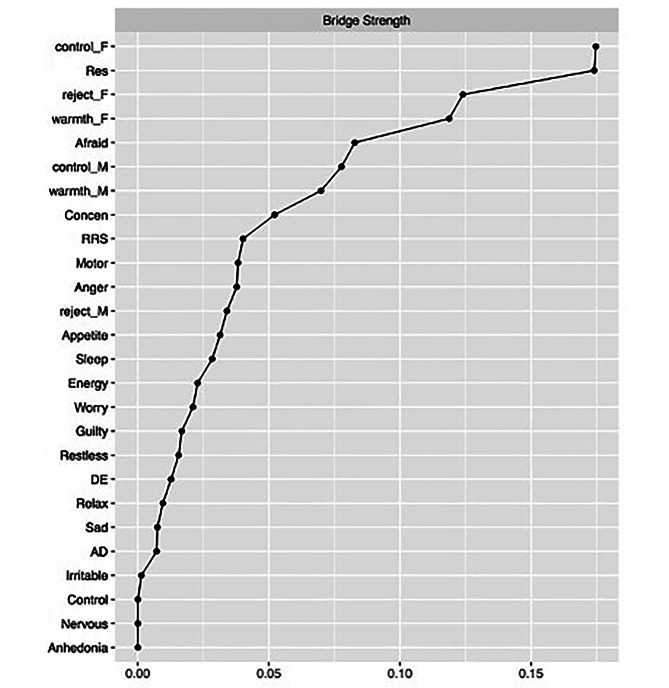
The bridge centrality indices of the variables in adolescent psychosocial network

Figure 2 shows the centrality indices of the bridge strength of each node. “Resilience”, “Afraid (afraid as if something would happen)”, “Concentration”, “Rumination”, and “Anger (shift from normal mood to anger)” exhibited the highest bridge strengths. Bridge strength stability, edge weights, and bridge centrality metrics were provided (see Figure S2-S4).

### Network associated with suicidal thoughts

The adolescent psychosocial network was re-estimated by adding a variable measuring active suicidal thoughts (see Fig. 1b). Estimated edge weights revealed that “Resilience” exhibited negative regularized edge weight with active suicidal thoughts (edge weights: -0.181, bootstrapped 95% CIs: -0.237 to -0.125). Furthermore, “AD (shift from anxiety to depression)” showed the strongest positive correlation with suicide (edge weights: 0.099, bootstrapped 95% CIs: 0.043 to 0.155), followed by “Anger”, “Rumination”, and “Anger (shift from normal mood to anger)” with edge weights (bootstrapped 95% CIs) ranging from 0.057 (0.001, 0.112) to 0.081 (0.026, 0.136). We did not find a significant correlation between parenting styles and active suicidal thoughts (see Table S4).

The results of NCT did not find significant differences in the global network strength (females:12.67, males:12.52, *p* = 0.15), network structure (maximum difference:0.12, *p* = 0.84), and edge weights across genders (see Figure S5).

Network plot incorporating covariates and the statistics of delta network was presented (see Figure S6 and Table S5). Overall, all the edge weights in the delta network are nearly zero, with the strongest edge weight of 0.07 in the network with active suicidal thoughts after controlling for covariates, suggesting that the covariates did not significantly factor into our network model.

### Linear regression analysis

Regression analysis showed that bridge strengths of proximal psychological factors in the adolescent psychosocial network were significantly correlated with their edge weights (absolute value) connecting active suicidal thoughts (R = 0.680, R^2^ = 0.432, *P* = 0.001, see Figure S7 and Table S6).

## Discussion

This study investigated the interplay between parenting styles and several proximal psychological factors associated with suicide in school-aged adolescents via network analysis. Results suggested that negative parenting styles may drive and maintain adolescent suicidal thoughts by activating some proximal psychological factors. Our study provides a further understanding of the psychopathology of adolescent suicidal thoughts and indicates that targeting specific bridge symptoms might deactivate adolescent suicide within the network.

First, we found that the proximal psychological variables (i.e., Resilience, Afraid, Concentration, Rumination, and Anger) have the highest bridge strengths in the adolescent psychosocial network, serving as bridge symptoms connecting clusters of parenting styles and proximal psychological factors. This observed relationship is consistent with previous longitudinal and meta-analytic findings on the association between adolescents’ emotional, cognitive, and behavioral problems and negative parenting styles [44, 45], It is acknowledged that parenting styles exert profound effects on shaping psychological development in childhood and adolescence [46, 47], and adolescents tend to respond with dysfunctional emotion or negative behavior when their emotional needs are not met by their parents during this critical and challenging period [48], Thus, negative parenting styles may act as a factor that increases the levels of rumination, anxiety, and depression and decreases adolescents’ resilience through a psychosocial network. However, further studies are needed to determine a causal relationship among the aforementioned relationships.

Notably, our results revealed that parenting styles did not have direct correlations with active suicidal thoughts; bridge symptoms of “Afraid”, “Rumination”, “AD” (shift from anxiety to depression)”, and “Anger (shift from normal mood to anger)”, were positively correlated with active suicidal thoughts; and “Resilience” was negatively associated with active suicidal thoughts in adolescents. Moreover, the identified bridge symptoms in the adolescent psychosocial network also have predictive power for indicating adolescent active suicidal thoughts over the past 24 h, with bridge strength explaining the 43.2% variance in the active suicidal thoughts. Our findings were in line with a previous study on patients with bipolar disorder exhibiting suicide attempts, which showed that, to some extent, anxiety symptoms were increased during the months prior to the onset of suicide attempts, suggesting that anxiety as a risk factor might contribute to the emergence of active suicidal thoughts [49], The bridge symptoms identified in our study may also be regarded as potential targets for prevention and treatment for reducing adolescent suicidal thoughts. As long as the bridge symptoms that served as risk factors were not activated or that served as protective factors were robust in the network, the adolescent suicidal risk would remain stable. Thus, future interventions may consider specifically focus on these bridge symptoms to help adolescents develop effective coping strategies when facing life changes and challenges.

Finally, although the network properties were not differed by gender, we identified that female adolescents reported not only a higher level of active suicidal thoughts than male adolescents, but also significantly higher levels of depression, anxiety, emotional lability, and a lower level of resilience than males. These findings were supported by a previous meta-analysis of longitudinal studies that females are more prone to exhibit internalizing problems such as mood disorders, whereas males are more likely to show higher risks of externalizing problems [50]. Besides, it’s worth noting that the perception of parenting styles neither differs by gender, except for male adolescents reporting a more heightened sense of rejection from fathers than female adolescents. Hence, it is necessary to consider the gender differences in psychological distress and rearing styles when developing tailored intervention strategies for adolescents. More further studies are needed to develop catered intervention strategies considering gender differences in the perception of parenting styles.

Several limitations exist in our study, and thus our findings should be interpreted with caution. First, given the cross-sectional design, we could not establish the temporal dynamic of the relationship between a set of proximal psychological factors and suicidal thoughts in adolescents, which future longitudinal cohort studies might settle. Second, adolescent suicidal thoughts were only measured by a self-report questionnaire assessing recent active suicidal thoughts rather than a systematic diagnosis and structural clinical assessment, which may lead to recall or response bias. Third, the current sample was recruited using convenient sampling, which are prone to selective bias. The adolescent sample in our study mostly from urban district and have relatively higher economic status family. However, parenting styles and parent-adolescent relationships might be primarily influenced by differences in cultures, regions, and socioeconomic statuses. Additionally, it’s worth considering that participants may have been more willing to engage in the study if they had specific concerns or experiences related to mental health. Therefore, the generalizations of our findings require more consideration due to the limited representativeness of our sample. Fourth, previous studies have indicated that psychiatric disorders were a major risk for suicidal behaviors [51]. Considering the lack of assessment of mental health conditions in our sample, future studies that are based on established clinical diagnosis for providing more actionable and optimizing prevention and intervention is needed. Last but not least, suicide in adolescents is a complex phenomenon, and various biological vulnerabilities and environmental stressors may contribute together to the emergence of adolescent suicidal thoughts and behaviors [19]. Future research that incorporates more comprehensive variables using a network model could be necessary.

## Conclusion

To our knowledge, this is the first network analysis to examine the interrelationships between parenting styles and a series of proximal psychological factors on the emergence of adolescent suicidal thoughts. Our findings indicate that the bridge symptoms connected with parenting styles and proximal psychological factors could be vital to understanding and alleviating adolescent suicidal thoughts. The network model may also expand our understanding of the complex mechanism of adolescent suicidal thoughts. Targeting specific bridge symptoms might have clinical implications for reducing adolescent suicide.

### Electronic supplementary material

Below is the link to the electronic supplementary material.

Supplementary Material 1

## Data Availability

The datasets used and analyzed during the current study are available from the corresponding author on reasonable request.
