# Supplementary material for: Bridge symptoms between parenting styles and proximal psychological risk factors associated with adolescent suicidal thoughts: a network analysis
Source: Child Adolesc Psychiatry Ment Health. 2023 Nov 15;17:129. doi: 10.1186/s13034-023-00674-z (PMC10652451; doi:10.1186/s13034-023-00674-z)
Supplement: Supplementary file 1 — Supplementary Material 1 [file 13034_2023_674_MOESM1_ESM.docx]

**Bridge symptoms between parenting styles and proximal psychological risk factors associated with adolescent suicidal thoughts: a network analysis**

***Supplemental Information***

**Network accuracy and stability analysis**

The accuracy of edge weights was evaluated by a non-parametric bootstrapping procedure, with smaller bootstrapped CIs indicating higher accuracy of the edge-weights. Stability of the centrality indices was evaluated by the case-dropping bootstrapping procedure. Various proportions of cases will be dropped from the dataset randomly in each bootstrap procedure, and the correlation was calculated between the original centrality indices and those obtained from subsets. To quantify the stability of centrality indices, we calculated correlation stability coefficient (CS-coefficient) by the maximum proportion of cases that can be dropped to retain a 95% bootstrapped CIs of correlation of ≥ 0.7 between original centrality indices and those from subsamples. Higher CS-coefficient indicates higher stability of the centrality indices. The CS-coefficient ≥ 0.25 indicates acceptable stability and ≥ 0.5 indicates good stability. In addition, the edge weights and centrality indices difference from one another were tested for significance by calculating the difference between bootstrap values of one edge-weight or centrality indices and another edge-weight or centrality indices. A zero in the bootstrapped CI indicates no significant difference between the edge-weights or centrality indices.

Supplementary Figure 1

Figure S1. Result of edge stability analyses in adolescent psychosocial network. The CS-coefficients for edge edge-weights is 0.75, Stability analyses revealed that bootstrapping CIs of most edge-weights were stable. Note: The x-axle indicates the edge weights and the y-axle indicates each edge. The black dots represent the mean value of the bootstrapped edge weights and the red dots represent the edge weights from the original sample. The gray area represented the 95% confidence intervals of the bootstrapped sample.

.

Supplementary Figure 2


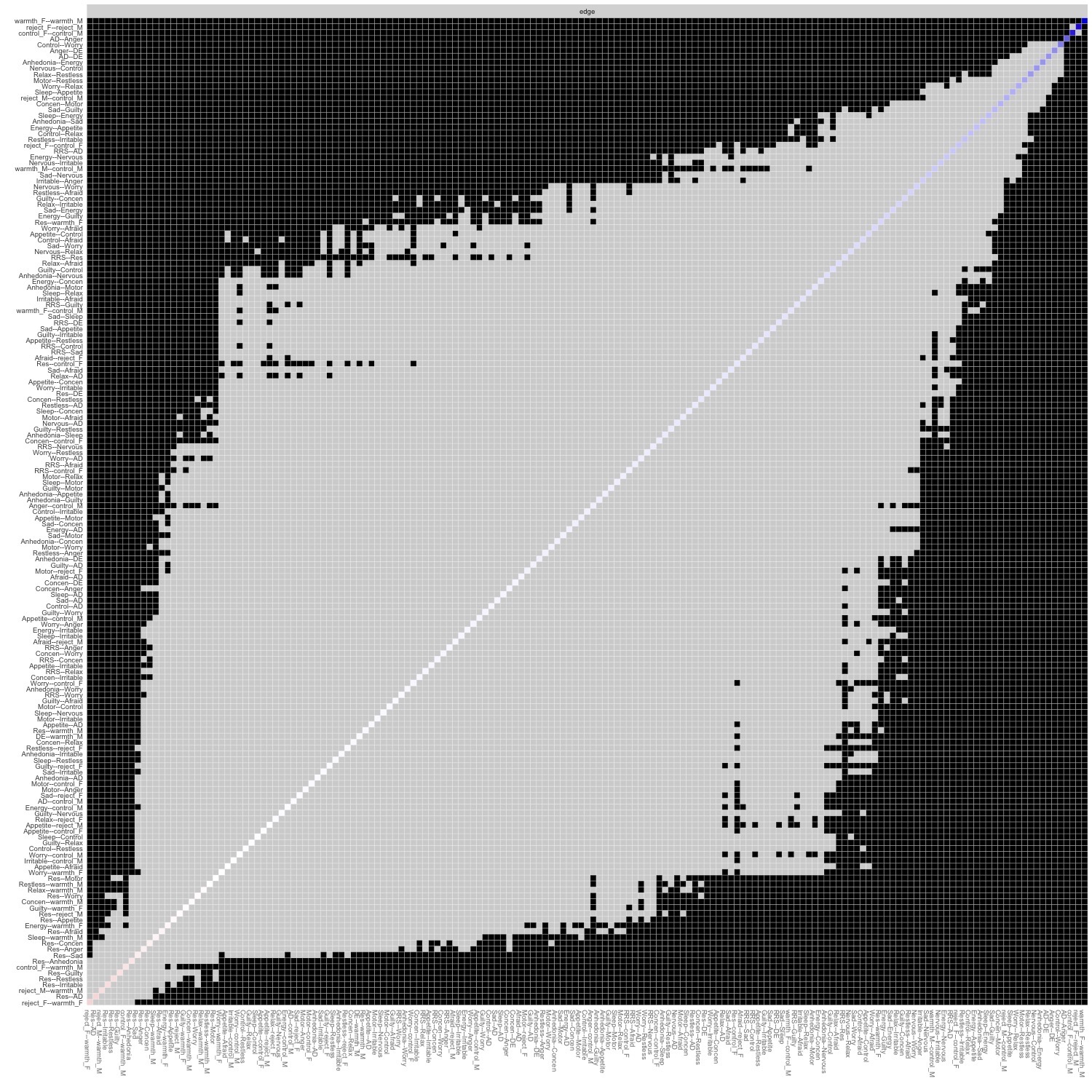


Figure S2. Result of difference tests for edge weight in adolescent psychosocial network

Supplementary Figure 3

Figure S3. Result of stability analyses on bridge strength in adolescent psychosocial network. The CS-coefficients for bridge strength is 0.67, respectively. Note: The x-axle indicates the proportion of cases included in the calculation of centrality indices, and the y-axle represents the correlation between the calculated centrality indices and the original centrality indices. The dots represent the mean value of the calculated centrality indices and the colored plates represent the 95% confidence intervals of the bootstrapped sample.

Supplementary Figure 4


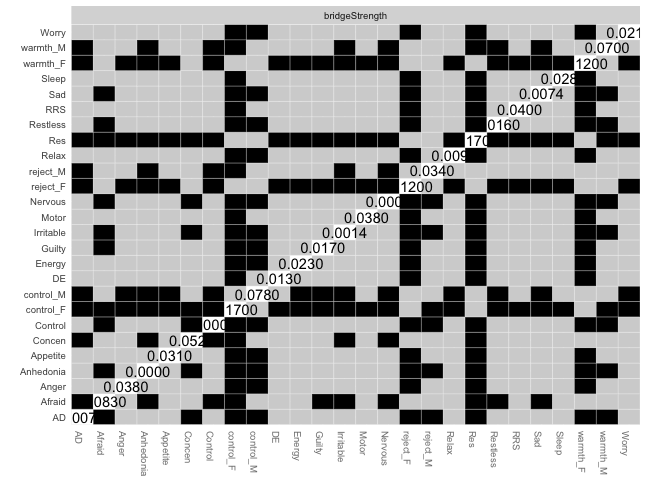


Figure S4. Result of difference tests for and bridge strength in adolescent psychosocial network

Supplementary Figure 5

A (Male)
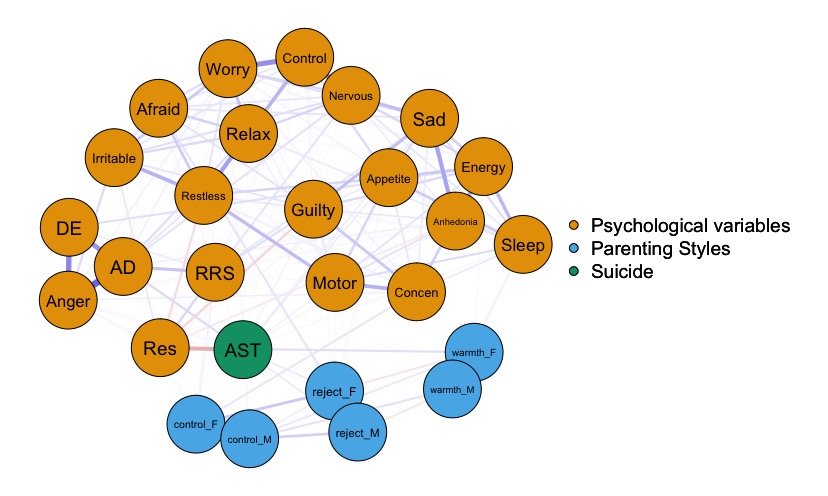


B (Female)


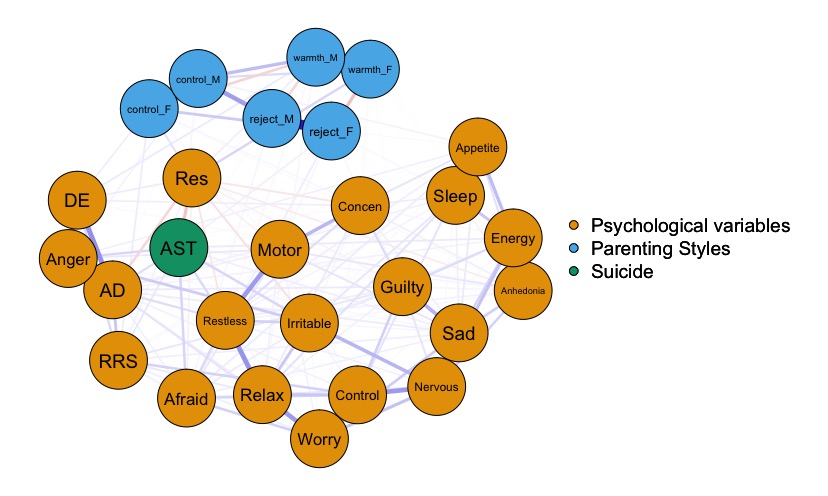


Fig S5. Estimated networks associated with suicidal thoughts in male (A) and female (B) participants

Supplementary Figure 6


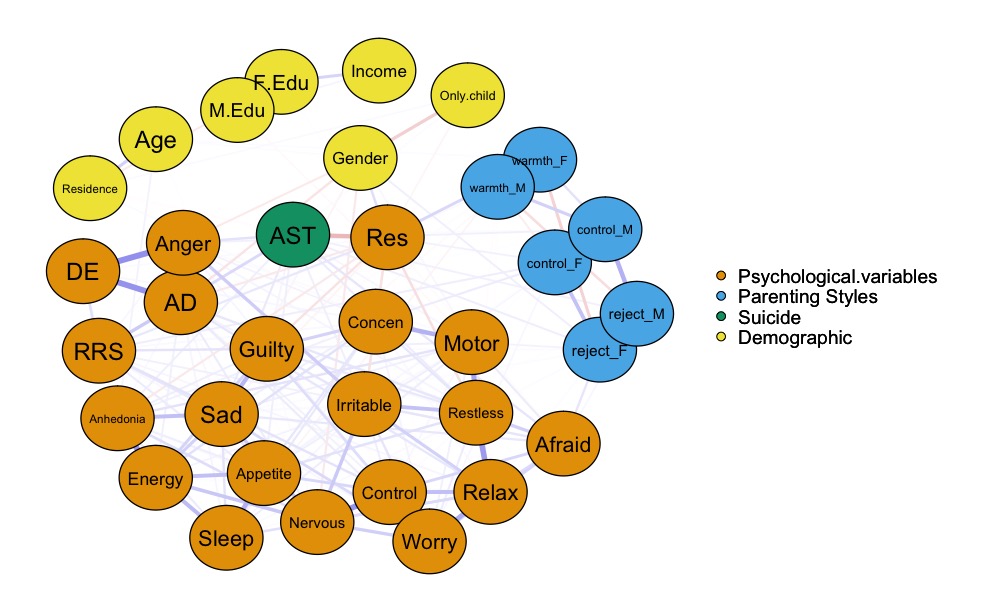


Figure S6. Estimated networks after controlling for covariates in network with suicidal thoughts. The color of nodes represents different symptom clusters, orange=Psychological variables, blue=Parenting styles, green=Active suicidal thoughts, yellow=Demographic; Node name and description: Psychological variables: Anhedonia= Anhedonia, Sad=Sad Mood, Sleep=Sleep, Energy =Energy, Appetite =Appetite, Guilty=Guilty, Concentration=Concentration, Motor=Motor, Nervous= Nervous , Control=Control Worry, Worry =Worry A Lot, Relax=Relax, Restless =Restless, Irritable=Irritable, Afraid=Afraid; Covariates: Age=Age; Sex=Sex; M.Edu=Educational level of mother,F.Edu=Educational level of father, Income=Income, Residence= Residence

Supplementary Figure 7
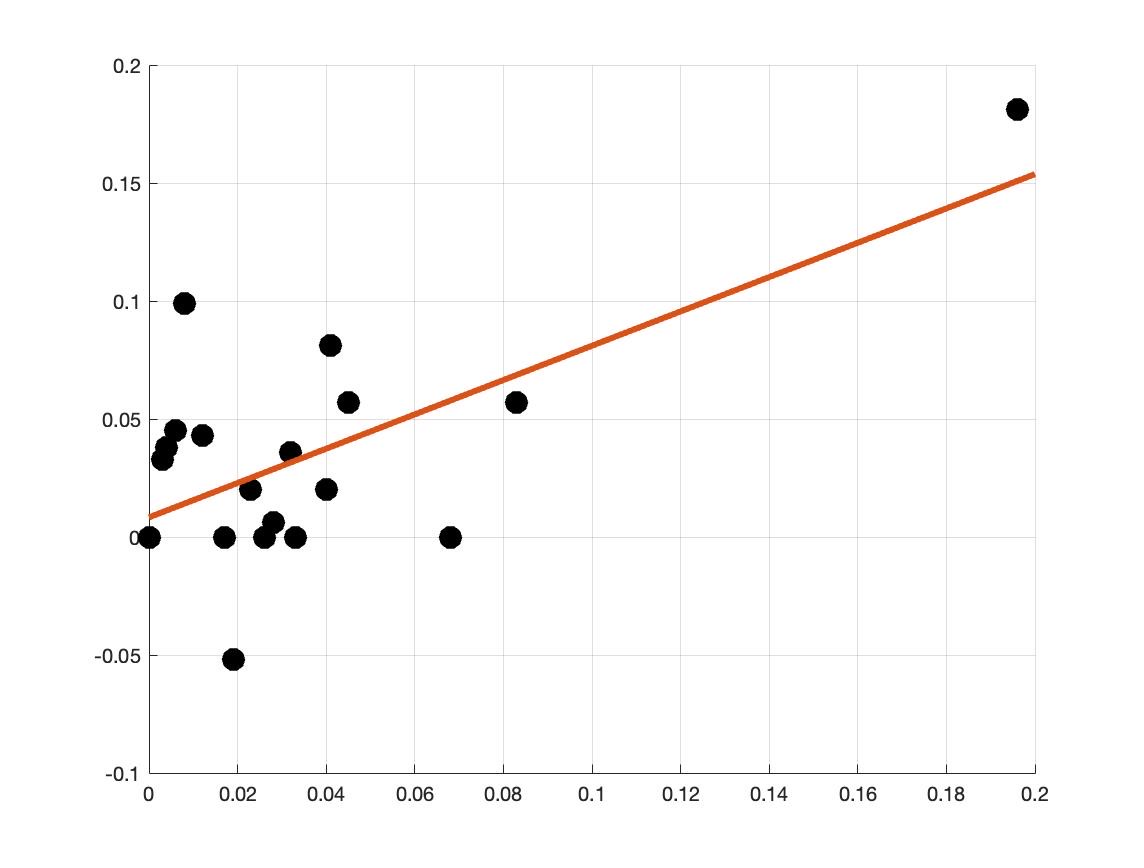


Figure S7. Result of linear regression analysis on the predictive utility of bridge symptoms for adolescent suicidal thoughts

Supplemental Table 1

Table S1. Gender Differences of all psychological variables

|  | Total  (N = 1117) | Male  (N = 511) | Female  (N = 660) | Z | *P* value |
| --- | --- | --- | --- | --- | --- |
| **Parental rearing** |  |  |  |  |  |
| Paternal Rejection (median, IQR) | 8 (8-9) | 8 (8-10) | 8 (8-9) | -2.300 | < 0.021 |
| Paternal Emotional Warmth (median, IQR) | 12.0 (12-16) | 12.0 (12-17) | 12.0 (12-16) | -0.811 | 0.418 |
| Paternal Overprotection (median, IQR) | 14.0 (13-14) | 14 (13-15) | 14 (13-14) | -1.850 | 0.064 |
| Maternal Rejection (median, IQR) | 8 (8-9) | 8 (8-10) | 8 (8-9) | -1.333 | 0.183 |
| Maternal Emotional Warmth (median, IQR) | 12(12-17) | 12 (13-15) | 12 (13-15) | -0.208 | 0.835 |
| Maternal Overprotection (median, IQR) | 13 (13-15) | 13 (12-17) | 13 (12-17) | -0.176 | 0.860 |
| **Resilience (median, IQR)** | 23 (19-28) | 24(19-29) | 21 (17-28) | -4.063 | < 0.001 |
| **Rumination (median, IQR)** | 30(25-37) | 33(27-40) | 29(24-34) | -6.740 | < 0.001 |
| **Affective lability (median, IQR)** |  |  |  |  |  |
| Anxiety/Depression | 5 (2-8) | 4 (0-6) | 6(3-9) | -8.504 | < 0.001 |
| Depression/Elation | 8 (3-11) | 7(1-10) | 8(5-11) | -6.055 | < 0.001 |
| Anger | 3 (0-4) | 2 (0-4) | 3(1-6) | -7.756 | < 0.001 |
| **PHQ-8 (median, IQR)** |  |  |  |  |  |
| Anhedonia | 1 (1-2) | 1 (0-1) | 1 (1-2) | -4.586 | < 0.001 |
| Sad Mood | 1 (1-1) | 1 (0-1) | 1 (1-2) | -6.491 | < 0.001 |
| Sleep | 1 (0-2) | 1 (0-1) | 1 (0-2) | -4.320 | < 0.001 |
| Energy | 1 (0-2) | 1 (0-2) | 1 (1-2) | -4.376 | < 0.001 |
| Appetite | 1 (0-1) | 0 (0-1) | 1 (1-2) | -4.617 | < 0.001 |
| Guilty | 1 (0-2) | 1 (1-2) | 1 (1-2) | -4.371 | < 0.001 |
| Centration | 1 (0-1) | 0 (0-1) | 1 (0-1) | -2.514 | 0.012 |
| Motor | 0 (0-1) | 0 (0-2) | 0 (0-2) | -0.381 | 0.703 |
| Total | 7 (4-10) | 6 (3-9) | 8 (5-10) | -5.955 | < 0.000 |
| **GAD-7 (median, IQR)** |  |  |  |  |  |
| Nervous | 1 (1-2) | 2 (0-1) | 1 (1-2) | -7.406 | < 0.001 |
| Bontrol Worry | 1 (0-1) | 1 (0-1) | 1 (0-2) | -5.536 | < 0.001 |
| Worry A Lot | 1 (0-2) | 1 (0-1) | 1 (0-2) | -4.431 | < 0.001 |
| Relax | 1 (0-1) | 0 (0-1) | 1 (0-1) | -3.533 | < 0.001 |
| Restless | 0 (0-1) | 0 (0-2) | 0 (0-2) | -2.004 | 0.045 |
| Irritable | 1 (0-1) | 1 (0-1) | 1 (0-2) | -5.091 | < 0.001 |
| Afraid | 1 (0-1) | 0 (0-0) | 1 (0-1) | -1.069 | 0.285 |
| Total | 5 (2-9) | 4 (1-7) | 6 (3-9.5) | -5.736 | < 0.001 |
| **Active suicidal thoughts (median, IQR) ^a^** | 6(3-9) | 3(3-8) **^b^** | 6(3-10) ^c^ | -4.635 | < 0.001 |

n, number of participants. a, obtained from 1158 participants. b, obtained from 508 male participants. c, obtained from 650 male participants

Supplemental Table 2

Table S2. Questionnaires and items used in psychological and behavior assessments

| Scale | Node Reference Name | Item (Abbreviation) | Item content |
| --- | --- | --- | --- |
| S-EMBU-C | Reject_F/ Reject_M | Rejection From Father/ Rejection From Mother | 1. My parents get angry with me without letting me know the reason.  4. My parents use physical punishment to discipline me.  7. My parents criticize me and tell me how lazy and useless I am in front of others.  13. I am treated as the ‘black sheep’ or ‘scapegoat’ of the family.  16. My parents treat me in such a way that I feel ashamed.  21. My parents punish me hard, even for small offenses. |
|  | Warmth_F/ Warmth_M | Emotional Warmth From Father/ Emotional Warmth From Mother | 2. My parents praise me.  6. My parents try to make my adolescence stimulating, interesting and instructive (ex. giving me good books, arranging for me to go to camps, taking me to sports/club activities).  12. My parents try to comfort and encourage me if things go badly for me.  14. My parents use words and gestures to show that they like me.  19. Warmth and tenderness exist between my parents and me.  23. My parents are proud when I succeed in something I have undertaken. |
|  | Control_F/  Control_M | Overprotection From Father/ Overprotection From Mother | 3. I wish my parents would worry less about what I am doing.  5. When I come home, I have to account for what I had been doing to my parents.  8. My parents forbid me to do things other adolescents are allowed to do because they are afraid that something might happen to me.  11. My parents get overly anxious that something might happen to me.  17. I am allowed to go wherever I like without my parents caring too much.  18. My parents interfere with everything I do.  22. My parents want to decide how I should dress or how I should look. |
| CD-RISC-10 | Res | Resilience | 1. I am able to adapt to change.  2. I can deal with whatever comes.  3. When I encounter problems, I can see the humorous side of things.  4. When I have to cope with stress, it strengthens me.  5. I tend to bounce back after illness, injury, or similar hardship.  6. I believe that I will achieve my goals even if there are obstacles.  7. I am not distracted under pressure and can think clearly.  8. I am not easily discouraged by failure.  9. I think of myself as a strong person dealing with life's difficulties.  10. I can handle unpleasant or painful feelings like sadness, fear, and anger. |
| RRS-10 | RRS | Rumination | 1.Think “What am I doing to deserve this?” |
|  |  |  | 2.Analyze recent events to try to understand why you are depressed |
|  |  |  | 3.Think “What do I always react this way?” |
|  |  |  | 4.Go away by yourself and think about why you feel this way |
|  |  |  | 5.Write down what you are thinking about and analyze it |
|  |  |  | 6.Think about a recent situation, wishing it had gone better |
|  |  |  | 7.Think “Why do I have problems other people don't have? |
|  |  |  | 8.Think “Why can't I handle things better?” |
|  |  |  | 9.Analyze your personality to try to understand why you are depressed |
|  |  |  | 10.Go someplace alone to think about your feelings |
| ASL-18 | AD | Anxiety/Depression | 1.At times I feel just as relaxed as everyone else and then within minutes I become so nervous that I feel light-headed and dizzy.  3. One minute I can be feeling OK and then the next minute I'm tense, jittery, and nervous.  5. Many times I feel very nervous and tense and then suddenly feel very sad and down.  6. Sometimes I go from feeling extremely anxious about something to feeling very down about it.  7. I shift back and forth from feeling perfectly calm to feeling uptight and nervous. |
|  | DE | Depression/Elation | 2.There are times when I have very little energy and then soon afterwards I have the same energy level as most people.  10. Sometimes I can think clearly and concentrate well one minute and then the next minute I have a great deal of difficulty concentrating and thinking clearly.  12. I switch back and forth between being extremely energetic and having so little energy that it's a huge effort just to get where I'm going.  13. There are times when I feel absolutely wonderful about myself but soon afterwards I often feel that I am just about the same as everyone else.  15. I shift back and forth between being very unproductive and being just as productive as everyone else.  16. Sometimes I feel extremely energetic one minute and then the next minute I might have so little energy that I can barely do a thing.  17. There are times when I have more energy than usual and more than most people and then soon afterwards I have about the same energy level as everyone else.  18. At times I feel that I'm doing everything at a slow pace but then soon afterwards I feel that I'm no more slowed down than anyone else. |
|  | Anger | Anger | 4. I frequently switch from being able to control my temper very well to not being able to control it very well at all.  8. There are times when I feel perfectly calm one minute and then the next minute the least little thing makes me furious.  9. Frequently, I will be feeling OK but then I suddenly get so mad that I could hit something.  11. There are times when I am so mad that I can barely stop yelling and other times shortly afterwards when I wouldn't think of yelling at all.  14. There are times when I'm so mad that my heart starts pounding and/or I start shaking and then shortly afterwards I feel quite relaxed. |
| PHQ-8 | Anhedonia | Anhedonia | 1. Little interest or pleasure in doing things |
|  | Sad | Sad Mood | 2. Feeling down, depressed, or hopeless |
|  | Sleep | Sleep | 3. Trouble falling or staying asleep, or sleeping too much |
|  | Energy | Energy | 4. Feeling tired or having little energy |
|  | Appetite | Appetite | 5. Poor appetite or overeating |
|  | Guilty | Guilty | 6. Feeling bad about yourself - or that you are a failure or have let yourself or your family down |
|  | Concen | Concentration | 7. Trouble concentrating on things, such as reading the newspaper or watching television |
|  | Motor | Motor | 8. Moving or speaking so slowly that other people could have noticed. Or so fidgety or restless that you have been moving a lot more than usual |
| GAD-7 | Nervous | Nervous | 1. Feeling nervous, anxious, or on edge |
|  | Control | Control Worry | 2. Not being able to stop or control worrying |
|  | Worry | Worry A Lot | 3. Worrying too much about different things |
|  | Relax | Relax | 4. Trouble relaxing |
|  | Restless | Restless | 5. Being so restless that it is hard to sit still |
|  | Irritable | Irritable | 6. Becoming easily annoyed or irritable |
|  | Afraid | Afraid | 7. Feeling afraid, as if something awful might happen |
| CHRT-SR | AST | Active suicidal thoughts | 1.I have been having thoughts of killing myself |
|  |  |  | 2.I have thoughts about how I might kill myself |
|  |  |  | 3.I have a plan to kill myself |

S-EMBU-C: short-form Egna Minnenav Barndoms Uppfostran for children; CD-RISC-10: 10-item Connor-Davidson Resilience Scale; RRS-10: 10-item rumination response scale; ASL-18: Affective Lability Scale-18; GAD-7: Generalized Anxiety Disorder Scale; PHQ-8: Patient Health Questionnaire-8; CHRT-SR:12-item concise health risk tracking self-report

Supplemental Table 3

Table S3. Estimated edge weights in the adolescent psychosocial network

|  | **RRS** | **Res** | **Anhedonia** | **Sad** | **Sleep** | **Energy** | **Appetite** | **Guilty** | **Concen** | **Motor** | **Nervous** | **Control** | **Worry** | **Relax** | **Restless** | **Irritable** | **Afraid** | **AD** | **Anger** | **DE** | **Reject_F** | **Warmth_F** | **Control_f** | **Reject_M** | **Warmth_M** | **Control_F** |
| --- | --- | --- | --- | --- | --- | --- | --- | --- | --- | --- | --- | --- | --- | --- | --- | --- | --- | --- | --- | --- | --- | --- | --- | --- | --- | --- |
| **RRS** | 0.00 | 0.08 | 0.00 | 0.06 | 0.00 | 0.00 | 0.00 | 0.07 | 0.02 | 0.00 | 0.04 | 0.06 | 0.02 | 0.02 | 0.00 | 0.00 | 0.04 | 0.13 | 0.02 | 0.07 | 0.00 | 0.00 | 0.04 | 0.00 | 0.00 | 0.00 |
| **Res** | 0.08 | 0.00 | -0.06 | -0.04 | 0.00 | 0.00 | -0.02 | -0.07 | -0.04 | 0.00 | 0.00 | 0.00 | -0.01 | 0.00 | -0.08 | -0.08 | -0.02 | -0.10 | -0.04 | 0.05 | 0.00 | 0.09 | 0.06 | -0.01 | 0.01 | 0.00 |
| **Anhedonia** | 0.00 | -0.06 | 0.00 | 0.16 | 0.05 | 0.26 | 0.04 | 0.04 | 0.03 | 0.07 | 0.08 | 0.00 | 0.02 | 0.00 | 0.00 | 0.01 | 0.00 | 0.01 | 0.00 | 0.03 | 0.00 | 0.00 | 0.00 | 0.00 | 0.00 | 0.00 |
| **Sad** | 0.06 | -0.04 | 0.16 | 0.00 | 0.07 | 0.10 | 0.06 | 0.17 | 0.04 | 0.03 | 0.13 | 0.00 | 0.09 | 0.00 | 0.00 | 0.01 | 0.06 | 0.02 | 0.00 | 0.00 | 0.01 | 0.00 | 0.00 | 0.00 | 0.00 | 0.00 |
| **Sleep** | 0.00 | 0.00 | 0.05 | 0.07 | 0.00 | 0.16 | 0.19 | 0.00 | 0.05 | 0.04 | 0.01 | 0.00 | 0.00 | 0.07 | 0.01 | 0.02 | 0.00 | 0.02 | 0.00 | 0.00 | 0.00 | 0.00 | 0.00 | 0.00 | -0.03 | 0.00 |
| **Energy** | 0.00 | 0.00 | 0.26 | 0.10 | 0.16 | 0.00 | 0.15 | 0.10 | 0.07 | 0.00 | 0.13 | 0.00 | 0.00 | 0.00 | 0.00 | 0.02 | 0.00 | 0.04 | 0.00 | 0.00 | 0.00 | -0.02 | 0.00 | 0.00 | 0.00 | 0.01 |
| **Appetite** | 0.00 | -0.02 | 0.04 | 0.06 | 0.19 | 0.15 | 0.00 | 0.00 | 0.06 | 0.04 | 0.00 | 0.09 | 0.00 | 0.00 | 0.06 | 0.02 | 0.00 | 0.01 | 0.00 | 0.00 | 0.00 | 0.00 | 0.00 | 0.00 | 0.00 | 0.02 |
| **Guilty** | 0.07 | -0.07 | 0.04 | 0.17 | 0.00 | 0.10 | 0.00 | 0.00 | 0.10 | 0.04 | 0.01 | 0.08 | 0.02 | 0.00 | 0.05 | 0.06 | 0.02 | 0.03 | 0.00 | 0.00 | 0.01 | -0.01 | 0.00 | 0.00 | 0.00 | 0.00 |
| **Concen** | 0.02 | -0.04 | 0.03 | 0.04 | 0.05 | 0.07 | 0.06 | 0.10 | 0.00 | 0.19 | 0.00 | 0.00 | 0.02 | 0.01 | 0.05 | 0.02 | 0.00 | 0.00 | 0.02 | 0.03 | 0.00 | 0.00 | 0.05 | 0.00 | -0.01 | 0.00 |
| **Motor** | 0.00 | 0.00 | 0.07 | 0.03 | 0.04 | 0.00 | 0.04 | 0.04 | 0.19 | 0.00 | 0.00 | 0.02 | 0.03 | 0.04 | 0.22 | 0.01 | 0.05 | 0.00 | 0.01 | 0.00 | 0.03 | 0.00 | 0.01 | 0.00 | 0.00 | 0.00 |
| **Nervous** | 0.04 | 0.00 | 0.08 | 0.13 | 0.01 | 0.13 | 0.00 | 0.01 | 0.00 | 0.00 | 0.00 | 0.25 | 0.11 | 0.08 | 0.00 | 0.13 | 0.00 | 0.05 | 0.00 | 0.00 | 0.00 | 0.00 | 0.00 | 0.00 | 0.00 | 0.00 |
| **Control** | 0.06 | 0.00 | 0.00 | 0.00 | 0.00 | 0.00 | 0.09 | 0.08 | 0.00 | 0.02 | 0.25 | 0.00 | 0.32 | 0.15 | 0.00 | 0.04 | 0.09 | 0.02 | 0.00 | 0.00 | 0.00 | 0.00 | 0.00 | 0.00 | 0.00 | 0.00 |
| **Worry** | 0.02 | -0.01 | 0.02 | 0.09 | 0.00 | 0.00 | 0.00 | 0.02 | 0.02 | 0.03 | 0.11 | 0.32 | 0.00 | 0.20 | 0.04 | 0.06 | 0.09 | 0.04 | 0.02 | 0.00 | 0.00 | 0.00 | 0.02 | 0.00 | 0.00 | 0.00 |
| **Relax** | 0.02 | 0.00 | 0.00 | 0.00 | 0.07 | 0.00 | 0.00 | 0.00 | 0.01 | 0.04 | 0.08 | 0.15 | 0.20 | 0.00 | 0.25 | 0.10 | 0.08 | 0.06 | 0.00 | 0.00 | 0.00 | 0.00 | 0.00 | 0.00 | 0.00 | 0.00 |
| **Restless** | 0.00 | -0.08 | 0.00 | 0.00 | 0.01 | 0.00 | 0.06 | 0.05 | 0.05 | 0.22 | 0.00 | 0.00 | 0.04 | 0.25 | 0.00 | 0.15 | 0.11 | 0.05 | 0.03 | 0.00 | 0.01 | 0.00 | 0.00 | 0.00 | 0.00 | 0.00 |
| **Irritable** | 0.00 | -0.08 | 0.01 | 0.01 | 0.02 | 0.02 | 0.02 | 0.06 | 0.02 | 0.01 | 0.13 | 0.04 | 0.06 | 0.10 | 0.15 | 0.00 | 0.07 | 0.00 | 0.12 | 0.00 | 0.00 | 0.00 | 0.00 | 0.00 | 0.00 | 0.00 |
| **Afraid** | 0.04 | -0.02 | 0.00 | 0.06 | 0.00 | 0.00 | 0.00 | 0.02 | 0.00 | 0.05 | 0.00 | 0.09 | 0.09 | 0.08 | 0.11 | 0.07 | 0.00 | 0.03 | 0.00 | 0.00 | 0.06 | 0.00 | 0.00 | 0.02 | 0.00 | 0.00 |
| **AD** | 0.13 | -0.10 | 0.01 | 0.02 | 0.02 | 0.04 | 0.01 | 0.03 | 0.00 | 0.00 | 0.05 | 0.02 | 0.04 | 0.06 | 0.05 | 0.00 | 0.03 | 0.00 | 0.40 | 0.26 | 0.00 | 0.00 | 0.00 | 0.00 | 0.00 | 0.01 |
| **DE** | 0.02 | -0.04 | 0.00 | 0.00 | 0.00 | 0.00 | 0.00 | 0.00 | 0.02 | 0.01 | 0.00 | 0.00 | 0.02 | 0.00 | 0.03 | 0.12 | 0.00 | 0.40 | 0.00 | 0.28 | 0.00 | 0.00 | 0.00 | 0.00 | 0.00 | 0.04 |
| **Anger** | 0.07 | 0.05 | 0.03 | 0.00 | 0.00 | 0.00 | 0.00 | 0.00 | 0.03 | 0.00 | 0.00 | 0.00 | 0.00 | 0.00 | 0.00 | 0.00 | 0.00 | 0.26 | 0.28 | 0.00 | 0.00 | 0.00 | 0.00 | 0.00 | 0.01 | 0.00 |
| **Reject_F** | 0.00 | 0.00 | 0.00 | 0.01 | 0.00 | 0.00 | 0.00 | 0.01 | 0.00 | 0.03 | 0.00 | 0.00 | 0.00 | 0.00 | 0.01 | 0.00 | 0.06 | 0.00 | 0.00 | 0.00 | 0.00 | -0.10 | 0.14 | 0.63 | 0.00 | 0.00 |
| **Warmth_F** | 0.00 | 0.09 | 0.00 | 0.00 | 0.00 | -0.02 | 0.00 | -0.01 | 0.00 | 0.00 | 0.00 | 0.00 | 0.00 | 0.00 | 0.00 | 0.00 | 0.00 | 0.00 | 0.00 | 0.00 | -0.10 | 0.00 | 0.00 | 0.00 | 0.76 | 0.07 |
| **Control_F** | 0.04 | 0.06 | 0.00 | 0.00 | 0.00 | 0.00 | 0.00 | 0.00 | 0.05 | 0.01 | 0.00 | 0.00 | 0.02 | 0.00 | 0.00 | 0.00 | 0.00 | 0.00 | 0.00 | 0.00 | 0.14 | 0.00 | 0.00 | 0.00 | -0.06 | 0.60 |
| **Reject_M** | 0.00 | -0.01 | 0.00 | 0.00 | 0.00 | 0.00 | 0.00 | 0.00 | 0.00 | 0.00 | 0.00 | 0.00 | 0.00 | 0.00 | 0.00 | 0.00 | 0.02 | 0.00 | 0.00 | 0.00 | 0.63 | 0.00 | 0.00 | 0.00 | -0.08 | 0.19 |
| **Warmth_M** | 0.00 | 0.01 | 0.00 | 0.00 | -0.03 | 0.00 | 0.00 | 0.00 | -0.01 | 0.00 | 0.00 | 0.00 | 0.00 | 0.00 | 0.00 | 0.00 | 0.00 | 0.00 | 0.00 | 0.01 | 0.00 | 0.76 | -0.06 | -0.08 | 0.00 | 0.13 |
| **control_M** | 0.00 | 0.00 | 0.00 | 0.00 | 0.00 | 0.01 | 0.02 | 0.00 | 0.00 | 0.00 | 0.00 | 0.00 | 0.00 | 0.00 | 0.00 | 0.00 | 0.00 | 0.01 | 0.04 | 0.00 | 0.00 | 0.07 | 0.60 | 0.19 | 0.13 | 0.00 |

Supplemental Table 4

Table S4. Estimated edge weights in symptom network associated with suicidal thoughts

|  | **RRS** | **Res** | **Anhedonia** | **Sad** | **Sleep** | **Energy** | **Appetite** | **Guilty** | **Concen** | **Motor** | **Nervous** | **Control** | **Worry** | **Relax** | **Restless** | **Irritable** | **Afraid** | **AD** | **Ang** | **DE** | **Reject_F** | **Warmth_F** | **Control_f** | **Reject_M** | **Warmth_M** | **Control_F** | **AST** |
| --- | --- | --- | --- | --- | --- | --- | --- | --- | --- | --- | --- | --- | --- | --- | --- | --- | --- | --- | --- | --- | --- | --- | --- | --- | --- | --- | --- |
| **RRS** | 0.00 | 0.13 | 0.00 | 0.06 | 0.00 | 0.00 | 0.00 | 0.08 | 0.02 | 0.00 | 0.04 | 0.06 | 0.01 | 0.02 | 0.00 | 0.00 | 0.04 | 0.13 | 0.02 | 0.07 | 0.00 | 0.01 | 0.04 | 0.00 | 0.00 | 0.00 | 0.06 |
| **Res** | 0.13 | 0.00 | -0.06 | -0.03 | 0.00 | 0.00 | -0.01 | -0.07 | -0.04 | 0.00 | 0.00 | 0.00 | -0.01 | 0.00 | -0.07 | -0.07 | -0.02 | -0.09 | -0.04 | 0.07 | 0.00 | 0.10 | 0.08 | -0.01 | 0.01 | 0.00 | -0.18 |
| **Anhedonia** | 0.00 | -0.06 | 0.00 | 0.15 | 0.05 | 0.27 | 0.04 | 0.04 | 0.03 | 0.07 | 0.08 | 0.00 | 0.01 | 0.00 | 0.00 | 0.01 | 0.00 | 0.00 | 0.00 | 0.04 | 0.00 | 0.00 | 0.00 | 0.00 | 0.01 | 0.00 | 0.04 |
| **Sad** | 0.06 | -0.03 | 0.15 | 0.00 | 0.06 | 0.11 | 0.06 | 0.17 | 0.03 | 0.03 | 0.13 | 0.00 | 0.09 | 0.00 | 0.00 | 0.00 | 0.05 | 0.02 | 0.00 | 0.00 | 0.00 | 0.00 | 0.00 | 0.00 | 0.00 | 0.00 | 0.05 |
| **Sleep** | 0.00 | 0.00 | 0.05 | 0.06 | 0.00 | 0.16 | 0.20 | 0.00 | 0.05 | 0.04 | 0.01 | 0.00 | 0.00 | 0.07 | 0.01 | 0.02 | 0.00 | 0.03 | 0.00 | 0.00 | 0.00 | 0.00 | 0.00 | 0.00 | -0.03 | 0.00 | 0.01 |
| **Energy** | 0.00 | 0.00 | 0.27 | 0.11 | 0.16 | 0.00 | 0.15 | 0.10 | 0.07 | 0.00 | 0.13 | 0.00 | 0.00 | 0.00 | 0.00 | 0.02 | 0.00 | 0.03 | 0.00 | 0.00 | 0.00 | -0.02 | 0.00 | 0.00 | 0.00 | 0.01 | 0.00 |
| **Appetite** | 0.00 | -0.01 | 0.04 | 0.06 | 0.20 | 0.15 | 0.00 | 0.00 | 0.06 | 0.04 | 0.00 | 0.09 | 0.00 | 0.00 | 0.07 | 0.02 | 0.00 | 0.00 | 0.00 | 0.00 | 0.00 | 0.00 | 0.00 | 0.01 | 0.00 | 0.03 | 0.04 |
| **Guilty** | 0.08 | -0.07 | 0.04 | 0.17 | 0.00 | 0.10 | 0.00 | 0.00 | 0.11 | 0.04 | 0.00 | 0.08 | 0.02 | 0.00 | 0.05 | 0.06 | 0.01 | 0.02 | 0.00 | 0.00 | 0.01 | -0.02 | 0.00 | 0.00 | 0.00 | 0.00 | 0.02 |
| **Concen** | 0.02 | -0.04 | 0.03 | 0.03 | 0.05 | 0.07 | 0.06 | 0.11 | 0.00 | 0.19 | 0.00 | 0.00 | 0.02 | 0.01 | 0.05 | 0.02 | 0.00 | 0.00 | 0.02 | 0.03 | 0.00 | 0.00 | 0.06 | -0.02 | -0.01 | 0.00 | 0.00 |
| **Motor** | 0.00 | 0.00 | 0.07 | 0.03 | 0.04 | 0.00 | 0.04 | 0.04 | 0.19 | 0.00 | 0.00 | 0.01 | 0.03 | 0.03 | 0.22 | 0.01 | 0.05 | 0.00 | 0.00 | 0.00 | 0.03 | 0.00 | 0.01 | 0.00 | 0.00 | 0.00 | 0.02 |
| **Nervous** | 0.04 | 0.00 | 0.08 | 0.13 | 0.01 | 0.13 | 0.00 | 0.00 | 0.00 | 0.00 | 0.00 | 0.26 | 0.11 | 0.08 | 0.00 | 0.13 | 0.00 | 0.05 | 0.00 | 0.00 | 0.00 | 0.00 | 0.00 | 0.00 | 0.00 | 0.00 | 0.00 |
| **Control** | 0.06 | 0.00 | 0.00 | 0.00 | 0.00 | 0.00 | 0.09 | 0.08 | 0.00 | 0.01 | 0.26 | 0.00 | 0.32 | 0.15 | 0.00 | 0.03 | 0.09 | 0.02 | 0.00 | 0.00 | 0.00 | 0.00 | 0.00 | 0.00 | 0.00 | 0.00 | 0.00 |
| **Worry** | 0.01 | -0.01 | 0.01 | 0.09 | 0.00 | 0.00 | 0.00 | 0.02 | 0.02 | 0.03 | 0.11 | 0.32 | 0.00 | 0.20 | 0.04 | 0.06 | 0.10 | 0.04 | 0.02 | 0.00 | 0.00 | 0.02 | 0.02 | 0.00 | 0.00 | 0.00 | 0.00 |
| **Relax** | 0.02 | 0.00 | 0.00 | 0.00 | 0.07 | 0.00 | 0.00 | 0.00 | 0.01 | 0.03 | 0.08 | 0.15 | 0.20 | 0.00 | 0.25 | 0.11 | 0.08 | 0.06 | 0.00 | -0.01 | 0.01 | 0.00 | 0.00 | 0.00 | -0.01 | -0.01 | 0.00 |
| **Restless** | 0.00 | -0.07 | 0.00 | 0.00 | 0.01 | 0.00 | 0.07 | 0.05 | 0.05 | 0.22 | 0.00 | 0.00 | 0.04 | 0.25 | 0.00 | 0.15 | 0.11 | 0.06 | 0.02 | 0.00 | 0.00 | 0.00 | 0.00 | 0.00 | -0.01 | 0.00 | 0.04 |
| **Irritable** | 0.00 | -0.07 | 0.01 | 0.00 | 0.02 | 0.02 | 0.02 | 0.06 | 0.02 | 0.01 | 0.13 | 0.03 | 0.06 | 0.11 | 0.15 | 0.00 | 0.06 | 0.00 | 0.12 | 0.00 | 0.00 | 0.00 | 0.00 | 0.00 | 0.00 | 0.01 | 0.03 |
| **Afraid** | 0.04 | -0.02 | 0.00 | 0.05 | 0.00 | 0.00 | 0.00 | 0.01 | 0.00 | 0.05 | 0.00 | 0.09 | 0.10 | 0.08 | 0.11 | 0.06 | 0.00 | 0.01 | 0.00 | 0.00 | 0.06 | 0.00 | 0.00 | 0.02 | 0.00 | 0.00 | 0.06 |
| **AD** | 0.13 | -0.09 | 0.00 | 0.02 | 0.03 | 0.03 | 0.00 | 0.02 | 0.00 | 0.00 | 0.05 | 0.02 | 0.04 | 0.06 | 0.06 | 0.00 | 0.01 | 0.00 | 0.40 | 0.28 | 0.00 | 0.00 | 0.00 | 0.00 | 0.00 | 0.00 | 0.10 |
| **DE** | 0.02 | -0.04 | 0.00 | 0.00 | 0.00 | 0.00 | 0.00 | 0.00 | 0.02 | 0.00 | 0.00 | 0.00 | 0.02 | 0.00 | 0.02 | 0.12 | 0.00 | 0.40 | 0.00 | 0.29 | 0.00 | 0.00 | 0.00 | 0.00 | 0.00 | 0.03 | 0.08 |
| **Ang** | 0.07 | 0.07 | 0.04 | 0.00 | 0.00 | 0.00 | 0.00 | 0.00 | 0.03 | 0.00 | 0.00 | 0.00 | 0.00 | -0.01 | 0.00 | 0.00 | 0.00 | 0.28 | 0.29 | 0.00 | 0.00 | 0.00 | 0.01 | -0.01 | 0.02 | 0.00 | -0.05 |
| **Reject_F** | 0.00 | 0.00 | 0.00 | 0.00 | 0.00 | 0.00 | 0.00 | 0.01 | 0.00 | 0.03 | 0.00 | 0.00 | 0.00 | 0.01 | 0.00 | 0.00 | 0.06 | 0.00 | 0.00 | 0.00 | 0.00 | -0.10 | 0.13 | 0.64 | 0.00 | 0.00 | 0.04 |
| **Warmth_F** | 0.01 | 0.10 | 0.00 | 0.00 | 0.00 | -0.02 | 0.00 | -0.02 | 0.00 | 0.00 | 0.00 | 0.00 | 0.02 | 0.00 | 0.00 | 0.00 | 0.00 | 0.00 | 0.00 | 0.00 | -0.10 | 0.00 | 0.00 | 0.00 | 0.77 | 0.07 | 0.00 |
| **Control_F** | 0.04 | 0.08 | 0.00 | 0.00 | 0.00 | 0.00 | 0.00 | 0.00 | 0.06 | 0.01 | 0.00 | 0.00 | 0.02 | 0.00 | 0.00 | 0.00 | 0.00 | 0.00 | 0.00 | 0.01 | 0.13 | 0.00 | 0.00 | -0.01 | -0.09 | 0.62 | 0.00 |
| **Reject_M** | 0.00 | -0.01 | 0.00 | 0.00 | 0.00 | 0.00 | 0.01 | 0.00 | -0.02 | 0.00 | 0.00 | 0.00 | 0.00 | 0.00 | 0.00 | 0.00 | 0.02 | 0.00 | 0.00 | -0.01 | 0.64 | 0.00 | -0.01 | 0.00 | -0.09 | 0.20 | 0.03 |
| **Warmth_M** | 0.00 | 0.01 | 0.01 | 0.00 | -0.03 | 0.00 | 0.00 | 0.00 | -0.01 | 0.00 | 0.00 | 0.00 | 0.00 | -0.01 | -0.01 | 0.00 | 0.00 | 0.00 | 0.00 | 0.02 | 0.00 | 0.77 | -0.09 | -0.09 | 0.00 | 0.16 | 0.00 |
| **Control_M** | 0.00 | 0.00 | 0.00 | 0.00 | 0.00 | 0.01 | 0.03 | 0.00 | 0.00 | 0.00 | 0.00 | 0.00 | 0.00 | -0.01 | 0.00 | 0.01 | 0.00 | 0.00 | 0.03 | 0.00 | 0.00 | 0.07 | 0.62 | 0.20 | 0.16 | 0.00 | 0.02 |
| **AST** | 0.06 | -0.18 | 0.04 | 0.05 | 0.01 | 0.00 | 0.04 | 0.02 | 0.00 | 0.02 | 0.00 | 0.00 | 0.00 | 0.00 | 0.04 | 0.03 | 0.06 | 0.10 | 0.08 | -0.05 | 0.04 | 0.00 | 0.00 | 0.03 | 0.00 | 0.02 | 0.00 |

Supplemental Table 5

Table S5. Estimated edge weights in symptom network associated with suicidal thoughts after controlling for covariates

|  | **RRS** | **Res** | **Anhedonia** | **Sad** | **Sleep** | **Energy** | **Appetite** | **Guilty** | **Concen** | **Motor** | **Nervous** | **Control** | **Worry** | **Relax** | **Restless** | **Irritable** | **Afraid** | **AD** | **Ang** | **DE** | **Reject_F** | **Warmth_F** | **Control_f** | **Reject_M** | **Warmth_M** | **Control_F** | **AST** |
| --- | --- | --- | --- | --- | --- | --- | --- | --- | --- | --- | --- | --- | --- | --- | --- | --- | --- | --- | --- | --- | --- | --- | --- | --- | --- | --- | --- |
| **RRS** | 0.00 | 0.07 | 0.00 | 0.01 | 0.00 | 0.00 | 0.00 | 0.01 | 0.01 | 0.00 | 0.00 | 0.00 | 0.00 | 0.00 | 0.00 | 0.00 | 0.01 | 0.01 | 0.00 | 0.00 | 0.00 | 0.07 | 0.00 | 0.01 | 0.00 | 0.00 | 0.00 |
| **Res** | 0.07 | 0.00 | -0.01 | -0.01 | 0.00 | 0.00 | 0.00 | -0.01 | -0.01 | 0.00 | 0.00 | 0.00 | -0.01 | 0.00 | 0.00 | 0.00 | -0.01 | -0.04 | -0.03 | 0.06 | 0.07 | 0.00 | -0.01 | -0.01 | 0.00 | 0.00 | 0.00 |
| **Anhedonia** | 0.00 | -0.01 | 0.00 | 0.00 | 0.00 | 0.01 | 0.00 | 0.00 | 0.00 | 0.00 | 0.00 | 0.00 | 0.00 | 0.00 | 0.00 | 0.00 | 0.00 | -0.01 | 0.00 | 0.02 | 0.00 | -0.01 | 0.00 | 0.00 | 0.00 | 0.01 | 0.00 |
| **Sad** | 0.01 | -0.01 | 0.00 | 0.00 | 0.00 | 0.00 | 0.00 | 0.01 | 0.00 | 0.00 | 0.00 | 0.00 | 0.00 | 0.00 | 0.00 | 0.00 | 0.00 | 0.00 | 0.00 | 0.00 | 0.01 | -0.01 | 0.00 | 0.00 | 0.00 | 0.00 | 0.00 |
| **Sleep** | 0.00 | 0.00 | 0.00 | 0.00 | 0.00 | 0.00 | 0.01 | 0.00 | 0.00 | 0.00 | 0.00 | 0.00 | 0.00 | 0.00 | 0.00 | 0.00 | 0.00 | 0.00 | 0.00 | 0.00 | 0.00 | 0.00 | 0.00 | 0.00 | 0.00 | 0.00 | 0.01 |
| **Energy** | 0.00 | 0.00 | 0.01 | 0.00 | 0.00 | 0.00 | 0.00 | 0.00 | 0.00 | 0.00 | 0.00 | 0.00 | 0.00 | -0.01 | 0.00 | 0.00 | 0.00 | 0.00 | 0.00 | 0.00 | 0.00 | 0.00 | 0.01 | 0.00 | 0.00 | 0.00 | 0.00 |
| **Appetite** | 0.00 | 0.00 | 0.00 | 0.00 | 0.01 | 0.00 | 0.00 | 0.00 | 0.00 | 0.00 | 0.00 | 0.00 | 0.00 | 0.00 | 0.00 | 0.00 | 0.00 | 0.00 | 0.00 | 0.00 | 0.00 | 0.00 | 0.00 | 0.00 | 0.01 | 0.00 | 0.00 |
| **Guilty** | 0.01 | -0.01 | 0.00 | 0.01 | 0.00 | 0.00 | 0.00 | 0.00 | 0.00 | 0.00 | 0.00 | 0.00 | 0.00 | 0.00 | 0.00 | 0.00 | 0.00 | 0.00 | 0.00 | 0.00 | 0.01 | -0.01 | 0.00 | 0.01 | 0.00 | 0.00 | 0.00 |
| **Concen** | 0.01 | -0.01 | 0.00 | 0.00 | 0.00 | 0.00 | 0.00 | 0.00 | 0.00 | 0.01 | 0.00 | 0.00 | 0.00 | 0.00 | 0.00 | 0.00 | 0.00 | 0.00 | -0.01 | 0.02 | 0.01 | -0.01 | 0.00 | 0.00 | 0.00 | 0.00 | 0.00 |
| **Motor** | 0.00 | 0.00 | 0.00 | 0.00 | 0.00 | 0.00 | 0.00 | 0.00 | 0.01 | 0.00 | 0.00 | 0.00 | 0.00 | 0.00 | 0.01 | 0.00 | 0.00 | 0.00 | 0.00 | 0.00 | 0.00 | 0.00 | 0.00 | 0.00 | 0.00 | 0.00 | 0.00 |
| **Nervous** | 0.00 | 0.00 | 0.00 | 0.00 | 0.00 | 0.00 | 0.00 | 0.00 | 0.00 | 0.00 | 0.00 | 0.01 | 0.00 | 0.00 | 0.00 | 0.00 | 0.00 | 0.00 | 0.00 | 0.00 | 0.00 | 0.00 | 0.00 | 0.00 | 0.00 | 0.00 | 0.00 |
| **Control** | 0.00 | 0.00 | 0.00 | 0.00 | 0.00 | 0.00 | 0.00 | 0.00 | 0.00 | 0.00 | 0.01 | 0.00 | 0.02 | 0.00 | -0.01 | 0.00 | 0.00 | 0.00 | 0.00 | 0.00 | 0.00 | 0.00 | 0.00 | 0.00 | 0.00 | 0.00 | 0.00 |
| **Worry** | 0.00 | -0.01 | 0.00 | 0.00 | 0.00 | 0.00 | 0.00 | 0.00 | 0.00 | 0.00 | 0.00 | 0.02 | 0.00 | 0.01 | 0.00 | 0.00 | 0.00 | 0.00 | 0.00 | 0.00 | 0.00 | -0.01 | 0.00 | 0.00 | 0.00 | 0.00 | 0.00 |
| **Relax** | 0.00 | 0.00 | 0.00 | 0.00 | 0.00 | -0.01 | 0.00 | 0.00 | 0.00 | 0.00 | 0.00 | 0.00 | 0.01 | 0.00 | 0.01 | 0.00 | 0.00 | 0.00 | 0.00 | -0.01 | 0.00 | 0.00 | 0.00 | 0.00 | 0.00 | -0.01 | 0.00 |
| **Restless** | 0.00 | 0.00 | 0.00 | 0.00 | 0.00 | 0.00 | 0.00 | 0.00 | 0.00 | 0.01 | 0.00 | -0.01 | 0.00 | 0.01 | 0.00 | 0.00 | 0.00 | 0.00 | 0.00 | 0.00 | 0.00 | 0.00 | 0.00 | 0.00 | 0.00 | 0.00 | 0.00 |
| **Irritable** | 0.00 | 0.00 | 0.00 | 0.00 | 0.00 | 0.00 | 0.00 | 0.00 | 0.00 | 0.00 | 0.00 | 0.00 | 0.00 | 0.00 | 0.00 | 0.00 | 0.00 | 0.00 | 0.00 | 0.00 | 0.00 | 0.00 | 0.00 | 0.00 | 0.00 | 0.00 | 0.00 |
| **Afraid** | 0.01 | -0.01 | 0.00 | 0.00 | 0.00 | 0.00 | 0.00 | 0.00 | 0.00 | 0.00 | 0.00 | 0.00 | 0.00 | 0.00 | 0.00 | 0.00 | 0.00 | 0.00 | 0.00 | 0.00 | 0.01 | -0.01 | 0.00 | 0.00 | 0.00 | 0.00 | 0.00 |
| **AD** | 0.01 | -0.04 | -0.01 | 0.00 | 0.00 | 0.00 | 0.00 | 0.00 | 0.00 | 0.00 | 0.00 | 0.00 | 0.00 | 0.00 | 0.00 | 0.00 | 0.00 | 0.00 | 0.01 | 0.03 | 0.01 | -0.04 | -0.01 | 0.00 | 0.00 | 0.00 | 0.00 |
| **DE** | 0.00 | -0.03 | 0.00 | 0.00 | 0.00 | 0.00 | 0.00 | 0.00 | -0.01 | 0.00 | 0.00 | 0.00 | 0.00 | 0.00 | 0.00 | 0.00 | 0.00 | 0.01 | 0.00 | 0.02 | 0.00 | -0.03 | 0.00 | 0.00 | 0.00 | 0.00 | 0.00 |
| **Ang** | 0.00 | 0.06 | 0.02 | 0.00 | 0.00 | 0.00 | 0.00 | 0.00 | 0.02 | 0.00 | 0.00 | 0.00 | 0.00 | -0.01 | 0.00 | 0.00 | 0.00 | 0.03 | 0.02 | 0.00 | 0.00 | 0.06 | 0.02 | 0.00 | 0.00 | 0.00 | 0.00 |
| **Reject_F** | 0.00 | 0.00 | 0.00 | 0.00 | 0.00 | 0.00 | 0.00 | 0.00 | 0.00 | 0.00 | 0.00 | 0.00 | 0.00 | 0.00 | 0.00 | 0.00 | 0.00 | 0.00 | 0.00 | 0.00 | 0.00 | 0.00 | 0.00 | 0.00 | 0.00 | 0.00 | 0.00 |
| **Warmth_F** | 0.01 | 0.00 | 0.00 | 0.00 | 0.00 | -0.01 | 0.00 | -0.01 | 0.00 | 0.00 | 0.00 | 0.00 | 0.02 | 0.00 | 0.00 | 0.00 | 0.00 | 0.00 | 0.00 | 0.00 | 0.01 | 0.00 | 0.00 | 0.00 | 0.00 | -0.01 | 0.00 |
| **Control_F** | 0.00 | 0.05 | 0.00 | 0.00 | 0.00 | 0.00 | 0.00 | 0.00 | 0.02 | 0.00 | 0.00 | 0.00 | 0.00 | 0.00 | 0.00 | 0.00 | 0.00 | 0.00 | 0.00 | 0.01 | 0.00 | 0.05 | 0.00 | 0.00 | 0.00 | 0.00 | 0.00 |
| **Reject_M** | 0.00 | -0.01 | 0.00 | 0.00 | 0.00 | 0.00 | 0.00 | 0.00 | -0.02 | 0.00 | 0.00 | 0.00 | 0.00 | 0.00 | 0.00 | 0.00 | 0.00 | 0.00 | 0.00 | -0.01 | 0.00 | -0.01 | 0.00 | 0.00 | 0.00 | 0.00 | 0.00 |
| **Warmth_M** | 0.00 | 0.00 | 0.01 | 0.00 | -0.01 | 0.00 | 0.00 | 0.00 | -0.01 | 0.00 | 0.00 | 0.00 | 0.00 | -0.01 | -0.01 | 0.00 | 0.00 | 0.00 | 0.00 | 0.02 | 0.00 | 0.00 | 0.01 | 0.00 | -0.01 | 0.00 | 0.00 |
| **Control_M** | 0.00 | 0.00 | 0.00 | 0.00 | 0.00 | 0.01 | 0.01 | 0.00 | 0.00 | 0.00 | 0.00 | 0.00 | 0.00 | -0.01 | 0.00 | 0.01 | 0.00 | 0.00 | 0.00 | 0.00 | 0.00 | 0.00 | 0.00 | 0.00 | 0.00 | 0.01 | 0.01 |
| **AST** | 0.02 | -0.01 | 0.00 | 0.00 | 0.00 | 0.00 | 0.00 | 0.00 | 0.00 | 0.00 | 0.00 | 0.00 | 0.00 | 0.00 | 0.00 | 0.00 | 0.00 | 0.01 | 0.01 | -0.05 | 0.02 | -0.01 | 0.00 | 0.00 | 0.00 | 0.00 | 0.00 |

Supplemental Table 6

Table S6. Bridge strengths of the proximal psychological factors and their edge weights connected to suicidal thoughts

|  | Brige strength | edge strength to suicidal thoughts |
| --- | --- | --- |
| RRS | 0.045 | 0.057 |
| RISC | 0.196 | -0.181 |
| Anhedo | 0.004 | 0.038 |
| Sad | 0.006 | 0.045 |
| Sleep | 0.028 | 0.006 |
| Energy | 0.026 | 0.000 |
| Appet | 0.032 | 0.036 |
| Guilty | 0.023 | 0.002 |
| Concen | 0.068 | 0.000 |
| Motor | 0.040 | 0.000 |
| Nervous | 0.000 | 0.000 |
| Control | 0.000 | 0.000 |
| Worry | 0.033 | 0.000 |
| Relax | 0.017 | 0.000 |
| Restless | 0.012 | 0.043 |
| Irritable | 0.003 | 0.033 |
| Afraid | 0.083 | 0.057 |
| AD | 0.008 | 0.099 |
| Anger | 0.041 | 0.081 |
| DE | 0.019 | -0.052 |
